# FUTURE PREOCCUPATION AND WELL-BEING: EXPANDING CONCEPTUALIZATION OF FUTURE TIME PERSPECTIVE

**DOI:** 10.1093/geroni/igad104.1820

**Published:** 2023-12-21

**Authors:** Li Chu, Tyler Matteson, Laura Carstensen

**Affiliations:** Stanford University, Stanford, California, United States; Stanford University, Stanford, California, United States; Stanford, Stanford, California, United States

## Abstract

Socioemotional selectivity theory posits that people prioritize emotionally meaningful goals as time horizons grow shorter and this shift in goals contributes to enhanced well-being. However, studies using the future time perspective (FTP) scale have yielded mixed findings regarding the association between time horizons and well-being. The present study aims to explore alternative constructs of time horizons to better understand well-being across the life span. In this preliminary study, a sample of 101 adults (age M = 46.99, SD = 19.20; 33 males, 66 females, and 2 indicated other gender identity) filled out an online questionnaire regarding time horizons and well-being. Exploratory and confirmatory factor analyses resulted in a new time horizon construct - time preoccupation, which captures the worries and fixations associated with future preparation. Serial mediation modeling shows the trend that people experience reduced future preoccupation with age, which contributes to more time savoring, and thus better well-being outcomes. Together, our findings suggest a promising role of future preoccupation in people’s well-being across the life span. These findings have important implications for future interventions that aim to improve younger adults’ well-being.

